# Exercise derived myokine irisin as mediator of cardiorespiratory, metabolic and thermal adjustments during central and peripheral chemoreflex activation

**DOI:** 10.1038/s41598-024-62650-7

**Published:** 2024-05-28

**Authors:** Mariana Bernardes-Ribeiro, Luis Gustavo A. Patrone, Caroline Cristina-Silva, Kênia C. Bícego, Luciane H. Gargaglioni

**Affiliations:** Departamento de Morfologia e Fisiologia Animal, Faculdade de Ciências Agrárias e Veterinárias, Universidade Estadual Paulista Júlio de Mesquita Filho (UNESP/FCAV), Via de Acesso Paulo Donato Castellane s/n, Jaboticabal, SP 14870-000 Brazil

**Keywords:** Myokine, Ventilation, Heart rate, Sleep, Respiration, Homeostasis

## Abstract

Exercise elicits physiological adaptations, including hyperpnea. However, the mechanisms underlying exercise-induced hyperpnea remain unresolved. Skeletal muscle acts as a secretory organ, releasing irisin (IR) during exercise. Irisin can cross the blood–brain barrier, influencing muscle and tissue metabolism, as well as signaling in the central nervous system (CNS). We evaluated the effect of intracerebroventricular or intraperitoneal injection of IR in adult male rats on the cardiorespiratory and metabolic function during sleep–wake cycle under room air, hypercapnia and hypoxia. Central IR injection caused an inhibition on ventilation (V_E_) during wakefulness under normoxia, while peripheral IR reduced V_E_ during sleep. Additionally, central IR exacerbates hypercapnic hyperventilation by increasing V_E_ and reducing oxygen consumption. As to cardiovascular regulation, central IR caused an increase in heart rate (HR) across all conditions, while no change was observed following peripheral administration. Finally, central IR attenuated the hypoxia-induced regulated hypothermia and increase sleep episodes, while peripheral IR augmented CO_2_-induced hypothermia, during wakefulness. Overall, our results suggest that IR act mostly on CNS exerting an inhibitory effect on breathing under resting conditions, while stimulating the hypercapnic ventilatory response and increasing HR. Therefore, IR seems not to be responsible for the exercise-induced hyperpnea, but contributes to the increase in HR.

## Introduction

In humans, during conditions of light or moderate physical exercise, partial pressures of blood gases change minimally, despite of the increase in metabolic rate as a result of physical activity^1,2^. Therefore, for more than a century there has been intense interest in the elucidation of how respiratory neurons adjust their activity, specially in response to physical exercise, since exercise hyperpnea is not associated with an increase in PaCO_2_ or a decrease in PaO_2_^3^. Despite the initial hypothesis that lactic acidosis would be the necessary stimulus to increase ventilation during intense exercise, Jeyaranjan et al.^4^ described that this stimulus is not an essential determinant of the exercise-induced hyperpnea. Further, as recently reviewed by Welch and Mitchell^5^, evidence suggests that both central and peripheral neurogenic factors contribute to exercise-induced hyperpnea. However, controversy surrounds these findings, and none of the proposed mechanisms, even when combined with feedback mechanisms, fully explain exercise hyperpnea. Therefore, other mediators, such as myokines, may be part of this signaling pathway between muscle activity and respiratory rhythm adjustments.

It is widely known that skeletal muscle generates antioxidant and hypoglycemic responses during physical activity^6^. The most plausible cellular explanations focus on the production of muscle cytokines, both by the endocrine system and paracrine action, the so-called myokines^7,8^. Irisin is a myokine composed by 112 amino acids, derived from a type 1 membrane protein present in myocytes and is encoded by the fibronectin type III domain containing 5 (FNDC5)^9,10^. More specifically, FNDC5 results in a protein formed by a signal peptide with 29 amino acids and has 100% similarity between rats and humans^11^. During physical activity, the contraction generated by muscles promotes activation of the adenosine monophosphate-activated protein kinase (AMPK) or p38 mitogen-activated protein kinase (p38MAPK) signaling pathways that stimulate the expression of proliferator-activated receptor gamma coactivator 1α (PGC-1α). The increase in PGC-1α in the cell drives the nucleus to a transcription process that will give rise to FNDC5. The production of IR occurs from enzymatic cleavage of FNDC5 carried out by the enzyme of the desintegrin and metalloproteinase family^12–14^.

Previous study has demonstrated that irisin, despite to be a large molecule, can cross the blood brain barrier (BBB)^15^. Irisin can stimulate the expression of the myokine brain-derived neurotrophic factor (BDNF) in the brain, an important signaling molecule for synaptic plasticity and neurogenesis^15^. Interestingly, lactate produced by the skeletal muscle resulting from exercise can stimulate the production of irisin in the brain in a PGC-1α-dependent manner^16^. Hence, given its widespread influence, irisin likely plays a role in facilitating the hyperpnea during exercise. However, no study has specifically assessed the involvement of this myokine in regulating respiration and the role of irisin in peripheral and central chemoreflex.

It is widely recognized that physical exercise, along with central and peripheral chemoreflex activation, not only affects ventilation promoting hyperpnea and enhancing CO_2_ and O_2_ chemosensitivity^17–19^, but also triggers significant cardiovascular, metabolic, and thermoregulatory adjustments^20,21^. Therefore, irisin may also play a role in regulating these adjustments. In fact, previous studies show that irisin converts white adipose tissue into brown adipose tissue, activating thermogenesis, mainly by upregulating the UCP1 expression through enhanced energy expenditure^9^. Since then, multiple other functions have been described such as that irisin stimulates neuroplasticity in the brain^22^, participates in cardiovascular control^23^, and has effects on sleep quality^24,25^.

In the present study, the experiments were undertaken to evaluate the participation of central and peripheral irisin in the cardiorespiratory and metabolic regulation under room air conditions, hypoxia and hypercapnia in adult rats. Since central respiratory control system is strongly influenced by vigilance state^26^, we compare the responses during sleep and wakefulness.

## Material and methods

### Animals

The experiments were performed in male adult Wistar rats, weighting 300–350 g. All the animals were housed in a temperature-controlled room, maintained at 25 ± 1 °C with a 12 h light–dark cycle (lights on at 6:30 a.m.), with water and food provided ad libitum. Experiments were performed between 7:00 a.m. and 6:00 p.m at the Department of Animal Morphology and Physiology of the Faculty of Agricultural and Veterinary Sciences (FCAV- UNESP, Jaboticabal campus). All protocols were approved by the local ethics committee called “Comissão de Ética no Uso de Animal” (CEUA—protocol no 3337/20), following the rules of the National Council for the Control of Animal Experimentation (CONCEA-Brazil). Furthermore, all experiments were performed in accordance with relevant guidelines and regulations and reporting in the manuscript follows the recommendations in the ARRIVE guidelines 2.0.

### Irisin treatment and gas mixture exposure

Irisin (Cayman Chemical, Ann Arbor, MI—EUA) was diluted in Tris buffer (0.05 M, pH ~ 8.0). This solution was used as vehicle for the control animals. We tested two concentrations of irisin, 0.425 and 1.66 μg/μL that correspond to the doses of 1.21 and 4.74 μg/kg respectively (based on the study of Zhang et al.^23,27^). Both concentrations were applied in the 4th brain ventricle (central; i.c.v.), whereas the higher concentration applied peripherally in different animals (peripheral i.p). The ventilatory challenges were accomplished using gas mixtures of hypercapnia (7% CO_2_, 21% O_2_, balance N_2_) and hypoxia (10% O_2_, balance N_2_) purchased from White Martins Gases Industrials Ltda (Osasco, SP, Brazil).

### Implantation of cannula in the 4th ventricle, electromyogram (EMG) and electroencephalogram (EEG)

One week prior to the experimental day, the rats were anesthetized with intraperitoneal injection of ketamine and xylazine (100 e 10 mg/kg, respectively). Then, the animals were fixed in a stereotaxic apparatus (Kopf Instruments, Kent, England) and a stainless-steel guide cannula (15 mm in length and 0.7 mm in external diameter) was implanted in the fourth ventricle, according to the coordinates adapted from the atlas of Paxinos and Watson^28^ (AP: − 11.9 mm from the bregma, LL: 0 mm, DV: − 6.7 mm from the skull). The cannula was fixed to the skull using dental acrylic and sealed with a mandrel to prevent occlusion and infection.

After this procedure, three electroencephalogram (EEG) electrodes were screwed into the skullcap: frontal electrode at 2 mm anterior to bregma and 2 mm lateral to the midline; parietal electrode at 4 mm anterior to the lambda and 2 mm lateral to the midline; and the third one, laterally between the frontal and parietal electrodes forming a triangle. For electromyogram (EMG) recordings, two electrodes were inserted deep into the neck musculature of the rats. All the electrodes were fixed to the animal’s head using a mini connector that was also soaked in acrylic cement. At the end, the animals were treated with antibiotic (enrofloxacin, 10 mg/kg, subcutaneous) and analgesic (flunixin meglumine, 2.5 mg/kg, subcutaneous) agents followed by the next 3 days and kept in cages up to two animals.

The signals from the EEG and EMG electrodes were sampled at 150 Hz, filtered at 0.3–50 and 0.1–100 Hz, respectively, and recorded on a computer with a data analysis software (AcqKnowledge MP150, BioPac Systems, Inc., Santa Barbara, CA, USA). Wakefulness, rapid eye movement (REM) or non-rapid eye movement (NREM) sleep states were registered constantly throughout the experiments. REM sleep periods were excluded from analysis since the events occurred shortly and irregularly between the experiments. The sleep/wake state was determined by analyzing the EEG and EMG records as previously described^29–31^, allowed cardiorespiratory and metabolic variables analyses during different phases of the sleep/wake cycle.

### Cardiovascular, blood gases and body temperature measurements

One day before the experiment, the rats received an intraperitoneal injection of ketamine and xylazine for another surgery procedure. This surgery consisted of insertion of a catheter [PE-10 connected to PE-50 (Clay Adams, Parsippany, NJ, USA)] into the abdominal aorta through the femoral artery, to allow pulsatile arterial pressure (PAP) and blood gases measurements. The catheter was externalized in the animal’s dorse close to the neck region. On the experiment day, the catheter was connected to the pressure transducer (TSD 104A, Biopac systems), the signal was amplified (DA 100C, Biopac systems) and digitized on a computer equipped with data acquisition software (MP100ACE; Biopac Systems). Systolic (SAP) and diastolic (DAP) arterial pressure, mean arterial pressure (MAP) and heart rate (HR) were quantified from the PAP recording using the LabChart program (Power-Lab System, ADInstruments®/Chart Software, version 7.3, Sydney, Australia).

For body temperature (T_B_) measurements, a temperature datalogger (SubCue Dataloggers, Calgary, Canada) was inserted into the abdominal cavity through a midline laparotomy, at the same surgical procedure. The datalogger was programmed to acquire data every 5 min.

### Central and peripheral treatments

For i.c.v., a Hamilton syringe (5 μL) and a needle (Mizzy, 200 μm of external diameter) where the tip was connected to a PE-10 polyethylene tube that connected to a 30 G gingival needle (15.5 mm long), was used to perform microinjections in the 4th ventricle. The volume of injections was 1 μL for 1 min using a microinjector machine (model 310, Stoelting Co., Il, EUA). For i.p. injections, the volume was established according to the animal’s body weight (1 mL/kg), and the injection was performed over a period of 15 s.

### Pulmonary ventilation assessment

Ventilation (V_E_) was recorded by using whole body plethysmography—closed system^32,33^. The chamber was sealed for approximately 2 min. The pressure oscillation due to temperature difference from inhaled and exhaled air were monitored by a differential pressure transducer (TSD 160A, Biopac Systems, Santa Barbara, CA, USA) and fed into a pre-amplifier (DA 100C, Biopac Systems), passed through an analog-to-digital converter and digitized on a computer equipped with data acquisition software (MP100ACE, Biopac Systems). The sampling frequency was 200 Hz. A volume calibration was performed by injecting 1 mL of air into the chamber using a graduated syringe. Tidal volume (V_T_) was calculated applying Drorbaugh and Fenn’s formula:^32^

$${\text{V}}_{{\text{T}}} = {\text{V}}_{{\text{K}}} \times \left( {{\text{P}}_{{\text{T}}} /{\text{P}}_{{\text{K}}} } \right) \times {\text{T}}_{{\text{B}}} \times \left( {{\text{P}}_{{\text{B}}} - {\text{P}}_{{\text{C}}} } \right)/{\text{T}}_{{\text{B}}} \times \left( {{\text{P}}_{{\text{B}}} - {\text{P}}_{{\text{C}}} } \right){-}{\text{T}}_{{\text{A}}} \times \left( {{\text{P}}_{{\text{B}}} {-}{\text{P}}_{{\text{R}}} } \right)$$

where P_T_: pressure deflection associated with each V_T_, P_K_: pressure deflection associated with the injection of the calibration volume (V_K_), T_A_: air temperature in the animal chamber, P_B_: barometric pressure, P_C_: water vapor pressure in the animal chamber, T_B_: body temperature (in Kelvin), and P_R_: water vapor pressure at T_C_. V_E_ was calculated as the product of respiratory frequency (f_R_) and V_T_ and divided to the animal’s body weight. V_E_ and V_T_ were presented under ambient barometric pressure conditions at T_B_ and saturated with water vapor (BTPS). P_C_ and P_R_ were calculated indirectly considering temperature and water vapor saturated air^34^. The LabChart software (PowerLab System, ADInstruments®/LabChart Software, version 7.3, Sydney, Australia) was used for data analysis.

### Determination of oxygen consumption

The indirect calorimetry method by measuring O_2_ consumption (VO_2_) with flow- through push mode configuration in an open respirometry system was used for metabolic rate inference^35,36^. The chamber air flow (2.2 L/min^−1^) was maintained through an aquarium pump that directed the air to the chamber entrance when the animals were exposed to ambient air and by gas mixture cylinders coupled to a flowmeter (model 822-13-OV1-PV2-V4, Sierra Instruments, Monterey, CA) when exposed to hypercapnia or hypoxia. The expired gas was dried over a small column of Drierite (W.A. Hammond Drierite Co. Ltd, Xenia, OH, USA) before entering the analyzer. The air was continuously sampled allowing for the determination of VO_2_ by a data acquisition program (Power-LabSystem, ADInstruments®/Chart Software, version 7.3, Sydney, Australia). CO_2_ was not analyzed or removed, then aiming at a better metabolic rate measurement the VO_2_ was calculated using the following equation:^37^

$${\text{VO}}_{2} = \left[ {{\text{FR}}_{{\text{e}}} \left( {{\text{F}}_{{\text{i}}} {\text{O}}_{2} {-}{\text{F}}_{{\text{e}}} {\text{O}}_{2} } \right)} \right]/\left[ {1{-}{\text{F}}_{{\text{i}}} {\text{O}}_{2} \left( {1{-}{\text{RQ}}} \right)} \right]$$

where FRe: end flow rate of air through the chamber, FiO_2_: inlet O_2_ fraction, FeO_2_: end O_2_ fraction, and RQ: respiratory quotient (considered to be 0.85). The VO_2_ was corrected for STPD (standard temperature pressure dry) and divided for body mass.

### Blood gases and pH measurements

Blood gases, pH and bicarbonate were measured in the following groups: i.c.v. or i.p.—Irisin and vehicle at room air, hypercapnia and hypoxia. Two drops of blood were sampled for immediate analyses of arterial pH (pHa), arterial carbon dioxide partial pressure (PaCO_2_), arterial oxygen partial pressure (PaO_2_) and plasma bicarbonate (HCO_3_^−^). These samples were transferred to an ECG8 + cartridge to be read in an i-STAT blood gas portable analyzer (i-STAT Analyzer, Abbott Laboratories, Libertyville Township, IL, USA).

### Experimental protocol

The animals were previously placed inside a plethysmography chamber and T_B_ was recorded in the data logger every 5 min. Initially, the chamber was ventilated with atmospheric air (21% O_2_), during a habituation time of 60 min. Then, the resting V_E_, VO_2_ e HR were measured in room air throughout 60 min. Following, the animals received an i.c.v. or i.p. injection of irisin or vehicle. After the injection, the animals were exposed to atmospheric air (21% O_2_), hypercapnia (7% CO_2_) or hypoxia (10% O_2_) during 60 min. For every condition of exposure, different animals were utilized. Ventilation was recorded for two minutes, during the baseline O_2_ recording, each 10 min during the exposure time. The arterial blood gases were made previously and 15 min after the microinjection. The VO_2_, HR, T_B_ measurements and EEG/EMG signals were recorded throughout the experiment.

### Determination of the heat loss index

In order to infer about the peripheral vasodilation or vasoconstriction of the animals that received the treatment with irisin or vehicle during exposure to hypoxia, we evaluated the heat loss vs conservation through the skin of the tail (T_S_). This protocol was performed according to Cristina-Silva et al.^38^. One week prior to the experimental day, the rats were anesthetized with i.p. injection of ketamine and xylazine (100 e 10 mg/kg, respectively) and were submitted to surgical procedure for the implantation of guide cannula in the 4th ventricle, as described above. One day before the experiment, the animals were anesthetized for the datalogger (SubCue Dataloggers, Calgary, Canada) implantation into the abdominal cavity through a midline laparotomy. The datalogger was programmed to acquire data every 1 min. The T_S_ and the ambient temperature (T_A_) were measured by using an infrared-sensitive camera (Flir SC660, Portland, OR, USA). The ambient temperature was maintained at ~ 25 °C and the animals were habituated in the chamber ventilated with atmospheric air (21% O_2_), during 30 min. Next, the thermographic camera was programed to take images under room air condition every 1 min during 30 min. Then, the microinjection (1 µL) of irisin 1.66 µg/µL or vehicle was performed through the guide cannula. After the injection, the animals were exposed to hypoxia (10% O_2_) during 30 min and imagens were taken at 1 min interval. Through thermographic images we were able to obtain the average of T_S_ and T_A_. The images during hypoxia were analyzed between 10 and 15 min, the peak moment of tail vasodilation caused by hypoxia^39^. Images were analysed using the Flir Tools® software (Flir Systems, OR, USA). The T_B_ and T_S_ were used to infer the heat loss index (HLI) of the animal, which ranges from 0 to 1 (where 0 indicates maximum vasoconstriction and 1 is maximum vasodilation), and was calculated according to the equation:^40^

$${\text{HLI}} = \left( {{\text{T}}_{{\text{s}}} - {\text{T}}_{{\text{A}}} } \right)/\left( {{\text{T}}_{{\text{B}}} - {\text{T}}_{{\text{A}}} } \right).$$

### Confirmation of the microinjection

At the end of the experiments, the animals were deeply anesthetized and received a microinjection of 1 μL of Evans blue solution (2%) via the central guide cannula to verify the location and distribution pattern of central microinjection. The animals were perfused through the left ventricle of the heart with 60 mL of sterile saline followed by 60 mL of 10% formalin. Then, the animals were decapitated, the brain was removed and sectioned for identification and confirmation of the microinjection site. Figure S1 displays images of a representative animal with a positive microinjection site on the 4th ventricle. Only the animals that showed blue coloration in this region were incorporated into the data analysis.

### Data analyses

Data are shown as the mean ± SEM. The effects of IR and vehicle on V_E_, f_R_, V_T,_ VO_2_, V_E_/VO_2_ and HR were analyzed using a one-way ANOVA and the animals were grouped according to sleep or wakefulness in each exposure condition. Approximately 2 min of recordings were used for respiratory (V_E_, f_R_, V_T_), metabolic (VO_2_) and cardiovascular (MAP, HR) measurements. None of the treatments altered MAP, therefore this data is not shown. Post-hoc multiple comparisons were performed using Tukey’s test when data were normally distributed to assess the differences among the treatments.

Data of blood gases, HLI, T_S_ and T_B_ were analyzed using two-way ANOVA. The analysis of sleep and wakefulness was conducted following the methodology outlined in the study by Vicente et al.^41^. Sleep and awake states were determined according to the pattern of EEG and EMG waves. Wakefulness was usually characterized by the EEG waves with low amplitude and high frequency and EMG with high activity. The sleep state was characterized by high amplitude and low frequency of EEG waves have and absence of EMG activity. EEG and EMG records were examined to determine the number of episodes, duration, and percentage of time spent by the animals in each state over a recording period of 3600 s. The comparison between the treatments and states was performed by two-way ANOVA repeated measures. The significance level adopted for all results was *p* < 0.05.

### Ethical approval

All protocols were approved by the local ethics committee called “Comissão de Ética no Uso de Animal” (CEUA—protocol no 3337/20), following the rules of the National Council for the Control of Animal Experimentation (CONCEA-Brazil). We confirm that the authors complied with the ARRIVE guidelines.

## Results

### Central treatment

#### Effect of central irisin microinjection on ventilation, metabolism, cardiovascular variables and body temperature under room air, hypercapnic and hypoxic conditions during wakefulness and sleep state

**Figure 1:**
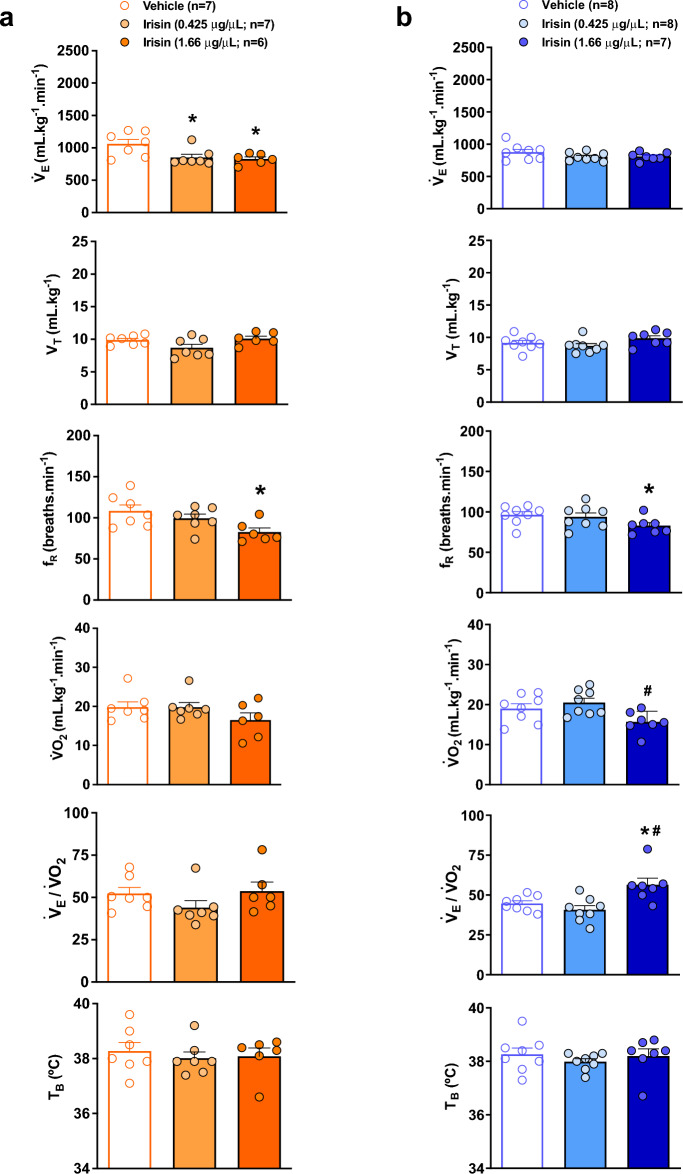
Effect of central irisin microinjection (0.425 or 1.66 µg/µL) on ventilation (V_E_), tidal volume (V_T_), respiratory frequency (f_R_), oxygen consumption (VO_2_), respiratory equivalent (V_E_/VO_2_) and body temperature (T_B_) during resting conditions in wakefulness (**a**) and sleep (**b**). Values are expressed as mean ± S.E.M. *Represents significant difference compared with control group. ^#^Represents significant difference between irisin-treated groups.

Figure 1 shows V_E_, V_T_, f_R_, VO_2_, V_E_/VO_2_ and T_B_ of control and irisin treated-animals (0.425 and 1.66 µg/µL) during wakefulness (Fig. 1a) and sleep (Fig. 1b) states while exposed to room air. During wakefulness, the central injection of both concentrations of irisin promoted a significant reduction in V_E_ under room air conditions (*P* < 0.02 for both concentrations), and the highest concentration also reduced the f_R_ (*P* < 0.02). No difference was observed in VO_2_ after the treatments. Regarding sleep state, the treatment with the highest IR concentration promoted a reduction in f_R_, differing from control animals (*P* < 0.03). Additionally, the treatment with the highest IR concentration caused a reduction in VO_2_ (*P* < 0.02) compared to the lowest concentration and, consequently, an increase in V_E_/VO_2_ compared to control and the lowest concentration groups (*P* < 0.02 and *P* < 0.01, respectively).

**Figure 2:**
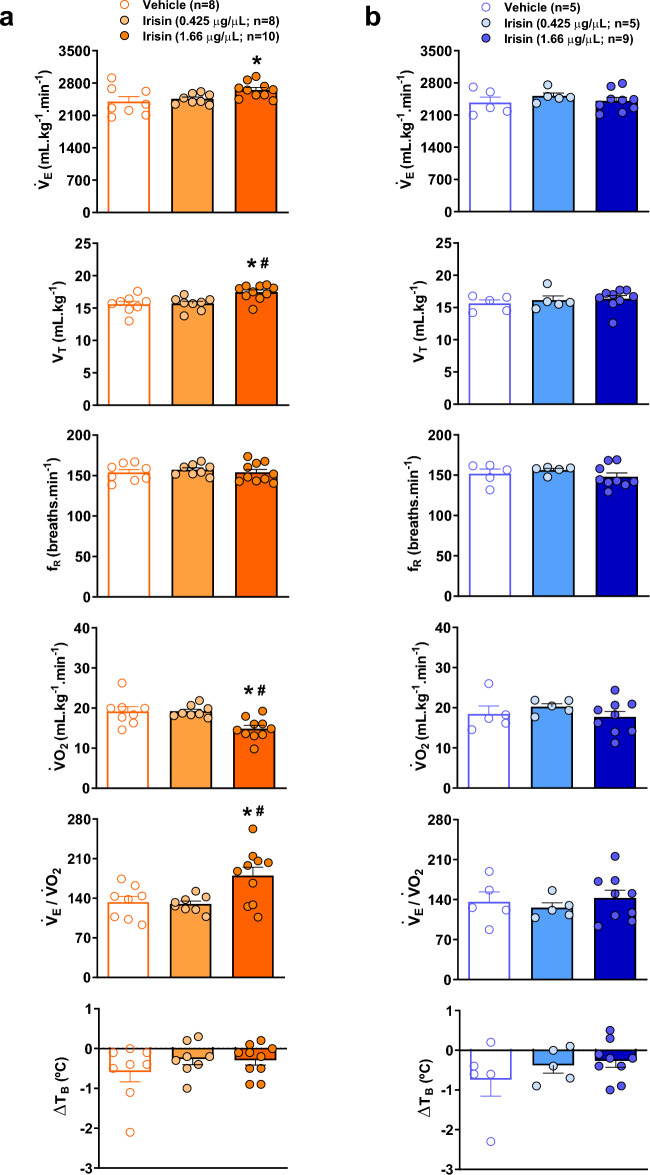
Effect of central irisin microinjection (0.425 or 1.66 µg/µL) on ventilation (V_E_), tidal volume (V_T_), respiratory frequency (f_R_), oxygen consumption (VO_2_), respiratory equivalent (V_E_/VO_2_) and body temperature (T_B_) during hypercapnia (7% CO_2_) in wakefulness (**a**) and sleep (**b**). Values are expressed as mean ± S.E.M. *Represents significant difference compared with control group. ^#^Represents significant difference between irisin-treated groups.

Figure 2 presents V_E_, V_T_, f_R_, VO_2_, V_E_/VO_2_ and T_B_ of control and irisin treated-animals during hypercapnia in the awake (Fig. 2a) and sleep (Fig. 2b) state. In wakefulness state, the central microinjection of the highest irisin concentration promoted an increase in V_E_ (*P* < 0.04) compared to the control hypercapnic animals. The effect was due to a significant change in V_T_ (*P* < 0.01 for control and *P* = 0.01 for IR 0.425 µg/µL). The highest concentration promoted a reduction in VO_2_ compared to control and the lowest concentration groups (*P* < 0.01 for both groups), as well as an increase in V_E_/VO_2_ (*P* < 0.03 and *P* < 0.02, respectively) leading to a hyperventilation. No significant differences were observed among treatments during sleep state, in the ventilatory, metabolic and T_B_ variables in hypercapnia.

**Figure 3:**
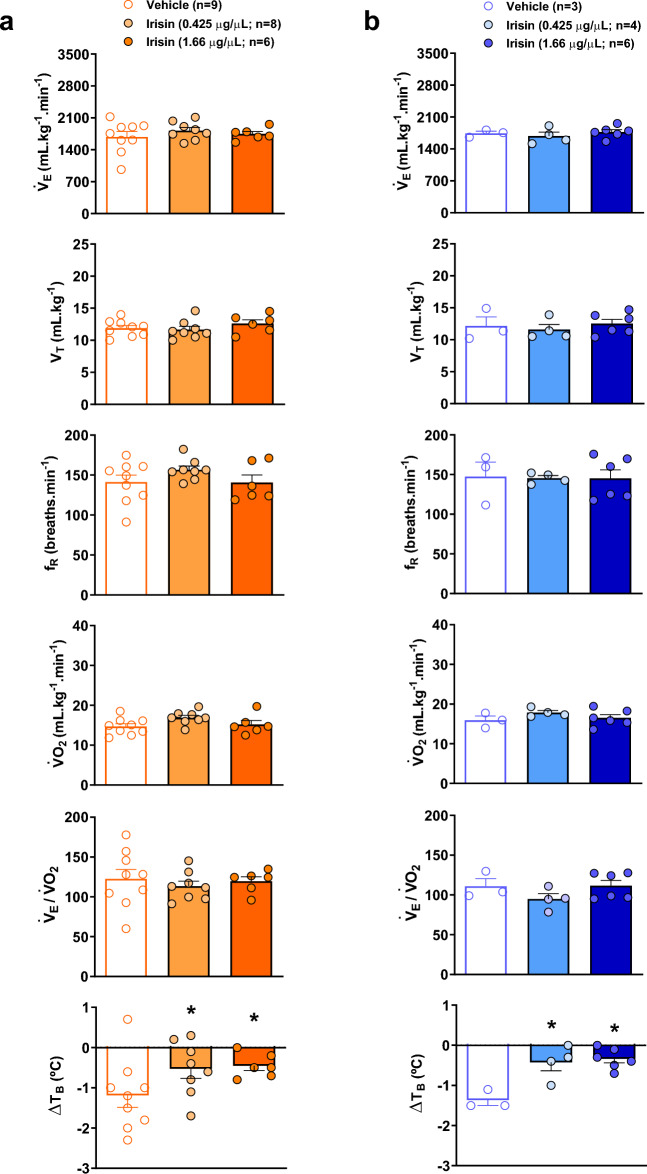
Effect of central irisin microinjection (0.425 or 1.66 µg/µL) on ventilation (V_E_), tidal volume (V_T_), respiratory frequency (f_R_), oxygen consumption (VO_2_), respiratory equivalent (V_E_/VO_2_) and body temperature (T_B_) during hypoxia (10% O_2_) in wakefulness (**a**) and sleep (**b**). Values are expressed as mean ± S.E.M. *Represents significant difference compared with control group.

Figure 3 demonstrates the V_E_, V_T,_ f_R_, VO_2,_ V_E_/VO_2_ and T_B_ of vehicle and irisin treated-animals during hypoxia in the awake (Fig. 3a) and sleep (Fig. 3b) state. Hypoxia caused a drop in T_B_ in the vehicle group, which was attenuated in the animals that received both IR concentrations during awake and sleep state (*P* < 0.05, for both treatments). The other variables were not affected by the IR treatments in hypoxia.

**Figure 4:**
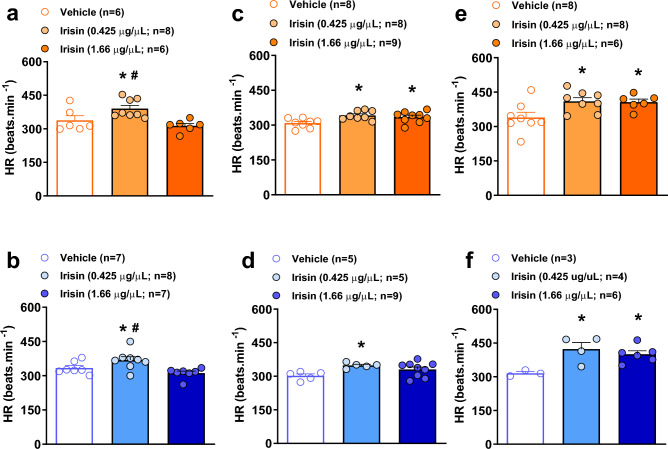
Effect of central irisin microinjection (0.425 or 1.66 µg/µL) on heart rate (HR) during wakefulness (**a**) and sleep (**b**) at room air condition, hypercapnia in wakefulness (**c**) and sleep (**d**) and hypoxia in awake (**e**) and sleep (**f**) states. Values are expressed as mean ± S.E.M. *Represents significant difference compared with control group within the same exposure condition. ^#^Represents significant difference between irisin-treated groups within the same exposure condition.

**Figure 5:**
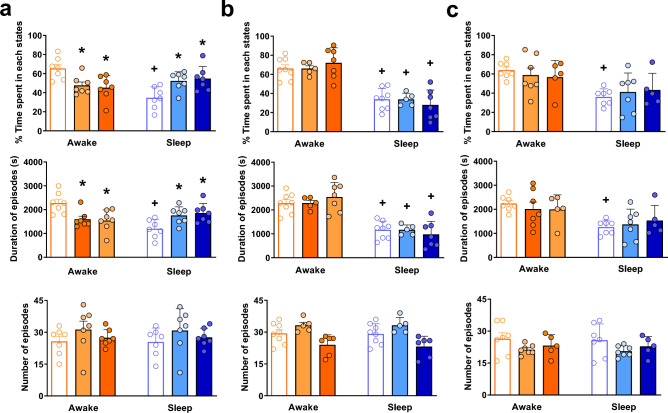
Effect of central irisin microinjection (0.425 or 1.66 µg/µL) on the percentage of time spent in each state, the duration of episodes (s) and the number of episodes during room air (**a**), hypercapnia (**b**) and hypoxia (**c**). Values are expressed as mean ± S.E.M. *Represents significant difference compared with control group within the same exposure condition. ^+^Means significant difference between awake and sleep state at the same treatment group and exposure condition.

Figure 4 presents the values of HR of the animals treated with irisin or vehicle in wakefulness. During room air conditions, the lowest IR concentration caused an increase in HR compared to control (*P* < 0.01 and *P* = 0.05, in awake and sleep states, respectively), and highest concentration (*P* < 0.01 for both awake and sleep states) (Fig. 4a, b). The microinjection of both IR concentrations resulted in a significant increase in HR (*P* = 0.01 for IR 0.425 µg/µL and *P* = 0.05 for IR 1.66 µg/µL) during wakefulness under hypercapnic challenge (Fig. 4c). Likewise, in sleep state, the lower IR concentration also promoted a significant increase in HR compared the control animals (*P* < 0.03, Fig. 4d). A similar pattern of response was observed under the hypoxic condition, during awake (Fig. 5e) and sleep (Fig. 5f) state. The treatment with both IR concentrations promoted an increase in HR compared to the control animals (*P* = 0.03, for both treatments in wakefulness; and *P* = 0.01 for IR 0.425 µg/µL and *P* = 0.05 for IR 1.66 µg/µL in sleep).

#### Effect of central microinjection of irisin on arterial pH and blood gases

Table 1 shows the effect of central irisin (0.425 or 1.66 µg/µL) microinjection on pHa, PaCO_2_, PaO_2_ and HCO_3_^−^ during room air conditions, hypercapnia and hypoxia. For all groups, exposure to 7% CO_2_ resulted in a decrease in pHa, increases in PaCO_2_, PaO_2_ and HCO_3_^-^ compared to room air conditions and hypoxia (*P* < 0.001, for all variables and conditions). Low environmental O_2_ condition caused an increase in pHa and a decrease in PaCO_2_ and PaO_2_ when compared to normoxia (*P* < 0.001, for all variables). Additionally, the animals treated with the highest IR concentration showed a significant increase in pHa and a decrease in the PaCO_2_ (*P* = 0.03 for both variables) during room air conditions, in addition to lower PaCO_2_ (*P* = 0.01), and HCO_3_^−^ (*P* < 0.02) when compared to the vehicle group during hypercapnia.

**Table 1:** Values of arterial pH (pHa), arterial carbon dioxide partial pressure (PaCO_2_), arterial oxygen partial pressure (PaO_2_) and plasma bicarbonate (HCO_3_^−^) 15 min after microinjection of vehicle or irisin (0.425 or 1.66 µg/µL) at room air, hypercapnic and hypoxic condition.

	Vehicle			Irisin (0.425 µg/µL)			Irisin (1.66 µg/µL)		
	Room air (n = 7)	Hypercapnia (n = 8)	Hypoxia (n = 7)	Room air (n = 7)	Hypercapnia (n = 7)	Hypoxia (n = 7)	Room air (n = 7)	Hypercapnia (n = 7)	Hypoxia (n = 5)
pHa	7.42 ± 0.01	7.33 ± 0.01^#+^	7.60 ± 0.01^#^	7.44 ± 0.01	7.32 ± 0.01^#+^	7.61 ± 0.01^#^	7.46 ± 0.01*	7.30 ± 0.01^#+^	7.60 ± 0.01^#^
*P*aCO_2_ (mmHg)	36.1 ± 1.8	52.2 ± 1.9^#+^	19.7 ± 1.2^#^	32.0 ± 1.0	47.7 ± 2.5^#+^	19.4 ± 1.2^#^	28.0 ± 1.6*	44.2 ± 3.2^#+^*	18.8 ± 1.6^#^
*P*aO_2_ (mmHg)	89.8 ± 3.4	118.6 ± 2.2^#+^	31.0 ± 1.2^#^	87.8 ± 2.2	123.6 ± 2.5^#+^	31.8 ± 1.4^#^	88.3 ± 1.7	117.1 ± 2.2^#+^	30.4 ± 1.7^#^
HCO_3_^−^	23.2 ± 1.5	27.3 ± 1.0^+^	19.4 ± 1.1	21.4 ± 0.5	24.5 ± 1.5^+^	19.5 ± 1.3	19.6 ± 1.0	22.2 ± 1.8*	18.3 ± 1.5

*Means significant difference between vehicle and irisin group at the same condition. ^#^Means significant difference when compared with room air at the same treatment group. ^+^Means significant difference between hypercapnic and hypoxic condition at the same treatment group.

#### Effect of central irisin microinjection on sleep/wakefulness state

Figure 5 shows the effect of central treatment of irisin in the percentage of time spent in each state, and in the duration and the number of episodes in sleep and wakefulness state. During normoxia normocapnia (Fig. 5a), the rats receiving vehicle treatment had lower percentage and length of the episodes in sleep (*P* < 0.01 for both variables). Regarding the IR-treated rats, both concentrations reduced the percentage of total wakefulness (*P* < 0.02 for both concentrations) and the length of the episodes in awake (*P* < 0.01 for both concentrations) compared to the control group. Therefore, treatment with the two IR concentrations promoted an increase in the percentage of total sleep (*P* < 0.02 for both concentrations) and the duration of episodes in sleep under room air condition (*P* < 0.05 for both concentrations). The CO_2_ exposure (Fig. 5b) reduced the percentage of sleep and the duration of episodes in sleep (*P* < 0.01 for both variables and groups), with no IR effects. Under hypoxic condition (Fig. 5c), the animals that received the vehicle treatment had a lower total percentage and length of the episodes of sleep (*P* = 0.03 for both variables) without any significant differences among the groups.

#### Effect of central microinjection of irisin on body temperature and heat loss index under hypoxic conditions

**Figure 6:**
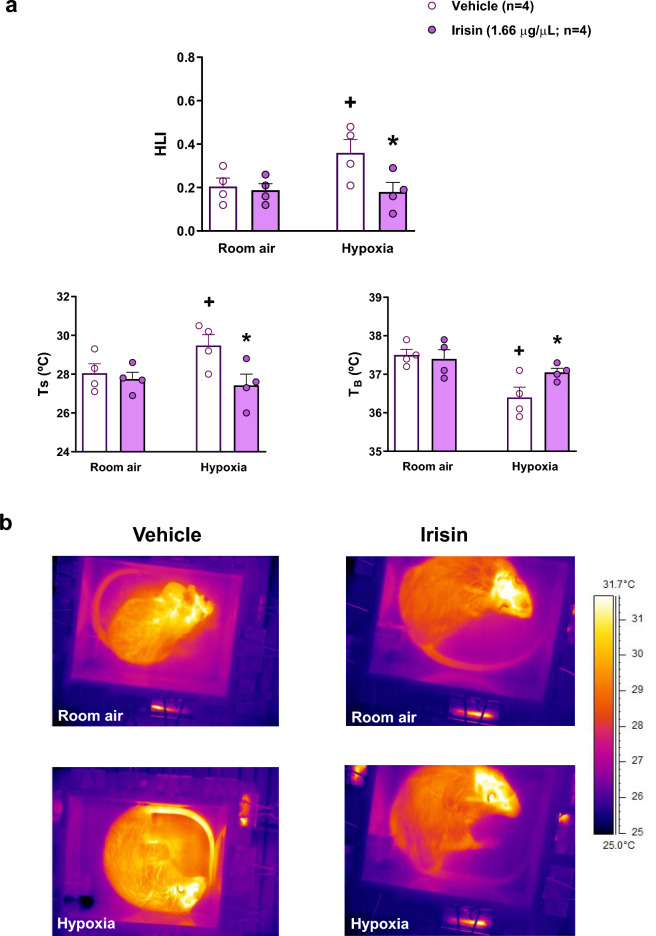
Effect of central irisin microinjection (1.66 µg/µL) on the heat loss index (HLI), tail skin temperature (Ts) and the body temperature (T_B_) during room air and hypoxia exposure (**a**). (**b**) Representative thermographic images of the animals in room air condition and after the microinjection of vehicle or irisin under hypoxia exposure.

For interpreting the attenuation of the hypoxia-induced regulated hypothermia by IR without change in VO_2_, we inferred changes in peripheral vasomotion by calculating the HLI from T_B_, T_A_ and T_S_ (Fig. 6). Figure 6a shows that hypoxia increased HLI in vehicle group (peripheral vasodilation) that occurred concurrently with the T_B_ drop and T_S_ increase. Central IR treatment during hypoxia inhibited the drop of T_B_ and the increase of T_S_ resulting in a lower HLI (Fig. 6a) compared to control animals (*P* < 0.01 for all variables), indicating peripheral vasoconstriction (Fig. 6b).

### Peripheral treatment

#### Effect of peripheral injection of irisin on ventilation, metabolism, cardiovascular variables and body temperature under room air, hypercapnic and hypoxic conditions during wakefulness and sleep state

Tables 2, 3 and 4 show the values of V_E_, V_T_, f_R_, VO_2_, V_E_/VO_2_, T_B,_ HR, the percentage of time spent in each state, in the duration and the number of episodes in sleep and wakefulness state during normoxia normocapnia (Table 2), hypercapnia (Table 3) and hypoxia (Table 4) of animals that received the i.p. treatment with irisin 1.66 µg/µL or vehicle in the awake and sleep states. Under room air conditions, IR treatment did not affect any of the measured variables (Table 2). During hypercapnia in wakefulness, the peripheral injection of IR decreased VO_2_ and, consequently, increased V_E_/VO_2_ compared to the control treatment (*P* < 0.03 for both variables) (Table 3). Additionally, the exposure to CO_2_ resulted in a greater decrease in T_B_ in IR-treated rats (*P* < 0.01). During sleep, IR treatment promoted an attenuation of hypercapnic ventilatory response due to a reduction in V_T_ compared to the vehicle group (*P* < 0.03 for both variables) (Table 3). As observed in wakefulness, IR treatment also potentiated the CO_2_-induced hipothermia during sleep (*P* < 0.04). The exposure to high levels of CO_2_ reduced the percetange of total sleep, the duration (*P* < 0.01 for both variables) and the number of episodes of sleep for control and IR animals (*P* < 0.03). During hypoxic challenge, peripheral IR treatment did not affect any variable (Table 4).

**Table 2:** Ventilation (V_E_), tidal volume (V_T_), respiratory frequency (f_R_), oxygen consumption (VO_2_), air convection requirement (V_E_/VO_2_), body temperature (T_B_), heart rate (HR), percentage of time spent in each state, duration and number of awake and sleep episodes for vehicle and irisin (1.66 µg/µL) peripherally treated animals at awake or sleep state under room air conditions.

	Awake		Sleep	
	Vehicle	Irisin (1.66 µg/µL)	Vehicle	Irisin (1.66 µg/µL)
V_E_ (mL kg^−1^ min^−1^)	808.9 ± 63.0	823.8 ± 36.6	780.7 ± 41.7	815.5 ± 45.6
V_T_ (mL kg^−1^)	8.8 ± 0.6	9.1 ± 0.3	9.1 ± 0.5	8.9 ± 0.2
f_R_ (breaths.min^-1^)	91.7 ± 3.9	90.2 ± 4.0	86.5 ± 4.0	91.1 ± 4.1
VO_2_ (mL kg^−1^ min^−1^)	16.1 ± 0.8	16.0 ± 0.7	16.4 ± 0.7	16.9 ± 0.5
V_E_/VO_2_	50.7 ± 3.7	51.0 ± 3.7	49.1 ± 3.8	49.0 ± 3.2
T_B_ (°C)	38.0 ± 0.3	38.2 ± 0.1	37.9 ± 0.2	38.2 ± 0.2
HR (beats min^−1^)	331.6 ± 9.1	350.8 ± 13.7	328.3 ± 7.6	356.3 ± 14.9
% Time spent in each states	51.4 ± 3.0	59.3 ± 4.5	48.6 ± 3.0	40.7 ± 4.5
Duration of episodes (s)	1797.5 ± 108.7	2113.6 ± 164.7	1700.8 ± 108.7	1448.6 ± 161.0
Number of episodes	23.6 ± 1.8	21.4 ± 1.7	23.1 ± 1.8	21.0 ± 1.7

**Table 3:** Ventilation (V_E_), tidal volume (V_T_), respiratory frequency (f_R_), oxygen consumption (VO_2_), air convection requirement (V_E_/VO_2_), body temperature (T_B_), heart rate (HR), percentage of time spent in each state, duration and number of awake and sleep episodes for vehicle and irisin (1.66 µg/µL) peripherally treated animals at awake or sleep state under hypercapnic conditions.

	Awake		Sleep	
	Vehicle	Irisin (1.66 µg/µL)	Vehicle	Irisin (1.66 µg/µL)
V_E_ (mL kg^−1^ min^−1^)	2465.7 ± 51.9	2361.2 ± 108.4	2435.3 ± 50.8	2198.0 ± 40.4*
V_T_ (mL kg^−1^)	15.9 ± 0.3	14.2 ± 1.1	15.6 ± 0.3	13.4 ± 0.6*
f_R_ (breaths min^−1^)	154.7 ± 4.0	168.2 ± 7.6	156.4 ± 4.4	165.9 ± 9.9
VO_2_ (mL kg^−1^ min^−1^)	20.0 ± 0.8	16.9 ± 1.0*	17.0 ± 1.3	15.4 ± 0.5
V_E_/VO_2_	125.9 ± 6.5	144.4 ± 6.5*	147.4 ± 10.9	146.0 ± 7.7
∆T_B_ (°C)	− 0.1 ± 0.1	− 0.6 ± 0.1*	− 0.3 ± 0.1	− 0.6 ± 0.1*
HR (beats min^−1^)	319.9 ± 9.8	324.1 ± 20.7	319.1 ± 7.1	324.6 ± 25.7
% Time spent in each states	70.2 ± 5.9	73.1 ± 7.0	29.8 ± 5.9^+^	26.8 ± 7.0^+^
Duration of episodes (s)	2544.1 ± 219.6	2562.0 ± 195.9	1075.6 ± 211.1^+^	965.0 ± 260.8^+^
Number of episodes	15.3 ± 0.7	14.6 ± 1.0	14.6 ± 0.8^+^	14.0 ± 1.2^+^

*Means significant difference between vehicle and irisin group at the same state of the sleep–wake cycle. ^+^Means significant difference between awake and sleep state at the same treatment group.

**Table 4:** Ventilation (V_E_), tidal volume (V_T_), respiratory frequency (f_R_), oxygen consumption (VO_2_), air convection requirement (V_E_/VO_2_), body temperature (T_B_), heart rate (HR), percentage of time spent in each state, duration and number of awake and sleep episodes for vehicle and irisin (1.66 µg/µL) peripherally treated animals at awake or sleep state under hypoxic conditions.

	Awake		Sleep	
	Vehicle	Irisin (1.66 µg/µL)	Vehicle	Irisin (1.66 µg/µL)
V_E_ (mL kg^−1^ min^−1^)	1634.1 ± 65.8	1566.4 ± 91.7	1606.7 ± 64.5	1507.7 ± 86.9
V_T_ (mL kg^−1^)	11.9 ± 0.6	11.5 ± 0.4	11.4 ± 0.6	11.1 ± 0.4
f_R_ (breaths min^−1^)	139.4 ± 7.1	136.1 ± 6.5	143.3 ± 7.3	137.5 ± 10.9
VO_2_ (mL kg^−1^ min^−1^)	15.5 ± 0.9	14.7 ± 0.9	15.4 ± 0.7	14.1 ± 0.3
V_E_/VO_2_	109.3 ± 7.0	112.0 ± 7	108.2 ± 3.3	108.5 ± 7.6
∆T_B_ (°C)	− 0.9 ± 0.1	− 0.7 ± 0.1	− 0.8 ± 0.2	− 1.0 ± 0.1
HR (beats min^−1^)	388.2 ± 15.7	367.0 ± 14.3	399.5 ± 27.9	358.9 ± 29.7
% Time spent in each states	55.3 ± 10.3	61.2 ± 7.5	44.6 ± 10.3	38.7 ± 7.5
Duration of episodes (s)	1865.0 ± 346.0	2266.0 ± 307.2	1532.0 ± 378.3	1413.0 ± 261.6
Number of episodes	17.4 ± 1.4	17.4 ± 1.6	16.8 ± 1.4	16.8 ± 1.7

#### Effect of peripheral injection of irisin on arterial pH and blood gases

Table 5 presents the effect of i.p. irisin (1.66 µg/µL) injection on pHa, PaCO_2_, PaO_2_ and HCO_3_^−^ during room air, hypercapnia and hypoxia conditions. Under room air conditions, peripheral IR did not cause alterations in blood gases, pHa or HCO_3_^−^. Exposure to 7% CO_2_ resulted in a decrease in pHa, increase in PaCO_2_ and PaO_2_ compared to room air conditions and hypoxia (*P* < 0.001, for all variables and conditions). Hypercapnia exposure also promoted an increase in HCO_3_^−^ compared to room air condition *(P* < 0.005, for the IR group) and hypoxia (*P* < 0.03 for the vehicle group; *P* < 0.001 for the IR group). Hypoxia promoted an increase in pHa and a decrease in PaCO_2_ and PaO_2_ when compared to normoxia (*P* < 0.001, for all variables) with no difference between groups. The peripheral injection of irisin did not promote significant difference in any of the conditions.

**Table 5:** Values of arterial pH (pHa), arterial carbon dioxide partial pressure (PaCO_2_), arterial oxygen partial pressure (PaO_2_) and plasma bicarbonate (HCO_3_^−^) 15 min after intraperitoneal injection of vehicle or irisin (1.66 µg/µL) at room air, hypercapnic and hypoxic condition.

	Vehicle			Irisin (1.66 µg/µL)		
	Room air (n = 5)	Hypercapnia (n = 6)	Hypoxia (n = 5)	Room air (n = 7)	Hypercapnia (n = 5)	Hypoxia (n = 6)
pHa	7.46 ± 0.01	7.33 ± 0.01^#+^	7.63 ± 0.01^#^	7.45 ± 0.01	7.34 ± 0.01^#+^	7.62 ± 0.01^#^
PaCO_2_ (mmHg)	29.9 ± 1.7	43.6 ± 1.6^#+^	17.7 ± 1.7^#^	30.0 ± 1.5	48.5 ± 1.8^#+^	17.4 ± 1.6^#^
PaO_2_ (mmHg)	81.8 ± 2.1	113.3 ± 1.9^#+^	31.4 ± 2.1^#^	80.8 ± 1.8	113.2 ± 2.3^#+^	31.6 ± 1.9^#^
HCO_3_^−^	21.3 ± 1.3	23.2 ± 1.2^+^	18.4 ± 1.3	21.0 ± 1.1	26.0 ± 1.4^#+^	18.0 ± 1.2

^#^Means significant difference when compared with room air at the same treatment group. ^+^Means significant difference between hypercapnic and hypoxic condition at the same treatment group.

## Discussion

Despite various studies conducted over the past century, the intricate mechanisms governing ventilatory control during exercise remain largely unresolved. Since exercise increases irisin secretion in skeletal muscles in humans and rodent species^41,42^, we hypothesized that IR may play a role in the augmentation of V_E_ and other physiological adaptations observed during physical activity. Our study demonstrates that IR, when acting on the CNS, exerts a tonic inhibitory effect on breathing control, promotes an increase in sleep period, and modulates heart rate in an excitatory manner. Therefore, it seems that this myokine does not account for the hyperpnea during exercise, but might be related to the cardiovascular adjustments, since an increase in HR was observed. Additionally, we have observed that both central and peripheral IR enhance CO_2_-induced hyperventilation during wakefulness which is also observed during acute exercise^19^. Furthermore, our findings provide compelling evidence of a diminished hypothermic response to hypoxia following central IR treatment, as indicated by a reduced HLI. Comparing the central and peripheral treatments with IR, lead us to suggest that the primary impacts of IR on the regulation of heart rate, ventilation, metabolic, and sleep/awake cycle occurs within the CNS.

Under resting conditions, central administration of IR led to a decrease in ventilation, particularly acting on f_R_, during wakefulness. This effect persisted during sleep, with a sustained impact on f_R_. Additionally, a decrease in VO_2_ was observed during sleep, ultimately culminating in a hyperventilatory response. The impact of the relative hyperventilation observed in central IR-treated rats was evident in the blood gas composition. Rats receiving the higher concentration of central IR exhibited a decrease in PaCO_2_ levels, leading to a subsequent increase in pHa. Therefore, in our study, it is possible that central IR is promoting a more efficient ventilatory response. Notably, peripheral IR treatment did not elicit any change in breathing control during resting conditions, indicating that its effect primarily occurs through the CNS. It is well established that during exercise, the elevation in pulmonary ventilation stems from increased f_R_ and V_T_, aimed at meeting the increased demands^3,43^. This must be accomplished efficiently, minimizing oxygen consumption by the respiratory system. Hence, our results contradict our initial hypothesis suggesting that IR could serve as a mediator of the hyperpneic response during exercise, since we observed a decreased V_E_ and f_R_ after IR injection.

During exposure to hypercapnia, central and peripheral treatments with IR promoted a potentiation of the hyperventilation during wakefulness mainly due to a reduction in VO_2_. In rats injected centrally, besides the alteration in VO_2_, an augment in the hypercapnic ventilatory response was observed due to an increase in V_T_. One possibility is that IR acts on the peripheral chemoreflex to enhance the CO_2_ ventilatory response. However, it is unlikely that this effect stems from an influence on the carotid body, as the hypoxic ventilatory response remained unaffected. Therefore, it is conceivable that IR administration might have influenced brainstem areas, leading to an augmented ventilatory response to CO_2_. In fact, both humans and rodents, FNDC5/irisin expression has been documented across various brain regions, including the hippocampus, midbrain, cerebellum, hypothalamus, cortex, and medulla oblongata^15,44^. Furthermore, irisin has been identified in the cerebrospinal fluid^15^. Interestingly, it is known that sensitivity to CO_2_ also increased during exercise^18,19,45^. According to Wright et al.^19^, in humans, the absence of a correlation between CO_2_ production and the hypercapnic ventilatory response implies that metabolic demand may not be the primary driving factor behind the heightened hypercapnic chemoreflex. The authors propose that the anticipation of exercise along with feedforward and mechanoreceptor feedback likely contribute to enhancing CO_2_ respiratory chemosensitivity. Nevertheless, additional studies are necessary to thoroughly investigate this aspect.

Administration of central IR resulted in an elevation in both the percentage of total sleep and the duration of sleep episodes at resting condition, suggesting that this myokine has the potential to enhance sleep quality. This effect could be attributed to a direct action of IR or the activation of BDNF^15^. A previous study has demonstrated that acute exercise induces the expression of BDNF in the hippocampus, and administering this factor into the rat brain during wakefulness can deepen subsequent non-REM sleep^25,46^. Furthermore, Kushikata et al.^47^ reported that administering BDNF by intracerebroventricular injection led to an increase in non-rapid eye movement sleep (NREMS) in both rats and rabbits. Subsequently, Faraguna et al.^46^ observed that unilateral cortical microinjections of BDNF in rats result in an augmentation of subsequent NREMS. Hence, it is plausible to think that the central treatment with IR induced an upregulation of BDNF expression, potentially contributing to the enhancement of sleep quantity during exposure to normal oxygen and carbon dioxide levels. Additionally, a study involving patients with rheumatoid arthritis, who often experience poor sleep quality, demonstrated lower serum irisin levels in comparison to patients with good sleep quality^24^.

Irisin was firstly discovered to play a role in the regulation of thermogenesis^9^. Previous study has demonstrated that central injection of irisin (1, 3 and 10 µM) causes an increase in body temperature in rats at 16, 24, and 48 h of interval after its application^48^. We did not observe changes in T_B_ in animals treated centrally with irisin or vehicle in both sleep or wake states during room air and hypercapnic conditions. However, peripheral IR treatment increased the hypothermic response under CO_2_ exposure in both states. It is well known that CO_2_ promotes peripheral vasodilation^49,50^ which could increase heat lost and, consequently, decrease T_B_. IR has also been shown to have a peripheral vasodilator effect^48^, which could potentiate the CO_2_ hypothermic response.

Interestingly, the animals that received i.c.v. injection of irisin at both concentrations (0.425 and 1.66 µg/µL) attenuated the drop of T_B_ during hypoxic exposure regardless sleep/wake state. The regulated hypothermia during hypoxia (normally called anapyrexia) is caused by an initial cutaneous vasodilation superimposed latter by thermogenesis inhibition^39,51–53^. As no effect on O_2_ consumption was observed, the irisin’s suppressive impact on regulated hypothermia in rats during hypoxia could be attributed to this myokine action on specific regions of the CNS involved in vasomotor control. IR could potentially activate areas responsible for sympathetic control, leading to peripheral vasoconstriction and heat conservation, effect that was accentuated during hypoxia. Notably, IR has been found to co-localize with neuropeptide Y in hypothalamic sections, specifically the paraventricular nucleus^54^. Moreover, a recent study conducted on zebrafish demonstrated some irisin effects on cardiovascular regulation and cardiac gene expression dependent on a sympathetic/beta-adrenergic pathway^55^. In the present study, we indeed showed that, in contrast to the vehicle-treated rats, central IR treated animals did not exhibit heat loss through the tail and did not present a significant decrease in T_B_, despite the fact they show no change in VO_2_. These results clearly indicate an inhibition by central IR of the hypoxia-induced peripheral vasodilation, thus, demonstrating the potential of IR acting on the CNS to modulate sympathetic activation, affecting heat conservation during adverse conditions such as hypoxia.

Similar to what happens during exercise^21^, central administration of IR at both concentrations increased HR during sleep and wakefulness states. However, there was no observed change in MAP in any of the conditions or treatments. According to Zhang et al.^23^, central IR administration increases cardiac output and blood pressure due to activation of neurons in the hypothalamic paraventricular nucleus. On the other hand, another study has reported that the microinjection of irisin into the nucleus ambiguus promotes a significant bradycardic response^56^. Considering the literature and our findings, the differential effects of IR on HR regulation could be attributed to several factors, including the varying concentrations administered, the specific region of microinjection, and the differences in animal models used. In a study conducted by Brailou et al.^56^, the effects of IR were evaluated in the nucleus ambiguus, a specific region involved in central parasympathetic cardiac control, where a bradycardic response was observed. In contrast, our study employed a more diffuse injection that likely reached different brainstem regions, including areas involved in central sympathetic cardiac control, such as the rostral ventrolateral medulla^57–59^. Consequently, the effects of IR on heart rate regulation may depend on the specific CNS region in which this peptide acts.

During exercise, a reduction in the tonic suppressive influence of parasympathetic (vagus) nerve activity contributes increases in HR, ventricular contractility, stroke volume, and thus, cardiac output^21^. Therefore, given that transcriptome analysis revealed high expression of IR in the medulla oblongata in mice^44^, where sympathetic and parasympathetic pre-motor neurons are situated, the HR elevation following central IR injection raises intriguing questions about the potential role of FNDC5/irisin in modulating autonomic cardiovascular regulation during exercise. However, further research is warranted to elaborate on these findings.

There are methodological considerations that warrant discussion. Firstly, we lack information on the extent to which peripherally injected IR crosses the blood–brain barrier, making it uncertain to affirm that the effect that we are observing is solely attributable to peripheral action. Therefore, future experiments should aim to quantify IR levels in the CNS following peripheral injection. Secondly, we did not conduct experiments to block IR release during exercise to assess its involvement in cardiorespiratory and metabolic control, thereby validating our hypothesis. Unfortunately, as far as we are aware, there are currently no pharmacological tools available in UpToDate to facilitate these experiments. Thirdly, the experiments were exclusively conducted in male rats, leaving uncertainty regarding the generalizability of the findings to females.

In conclusion, our data strongly suggest that the predominant influence of IR on cardiorespiratory regulation occurs through its action in the CNS. Central treatment with IR with the two concentrations generated a condition of hypoventilation under room air condition due to a lower V_E_ indicating an inhibitory role on breathing control, suggesting that this myokine seems not to be responsible for the exercise-induced hyperpnea. Increasing the concentration of IR potentiated the hyperventilatory response to CO_2_, promoting an increase in V_E_ and inhibiting the VO_2_ during wakefulness. In addition, this myokine increased HR and was capable of attenuating the drop in T_B_ under conditions of low O_2_ levels, which suggest a sympathetic excitatory role of IR. Thus, our results establish IR as a potent bioactive molecule. It also provides the basis for future research on the mechanisms of action of IR, and its role in other physiological processes in rats.

### Supplementary Information

Supplementary Information.

## Data Availability

All data that support the finding of the current study are available from LHG, upon reasonable request.
