# Supplementary material for: Exercise derived myokine irisin as mediator of cardiorespiratory, metabolic and thermal adjustments during central and peripheral chemoreflex activation
Source: Sci Rep. 2024 May 28;14:12262. doi: 10.1038/s41598-024-62650-7 (PMC11133352; doi:10.1038/s41598-024-62650-7)
Supplement: Supplementary file 1 — Supplementary Information. [file 41598_2024_62650_MOESM1_ESM.pdf]

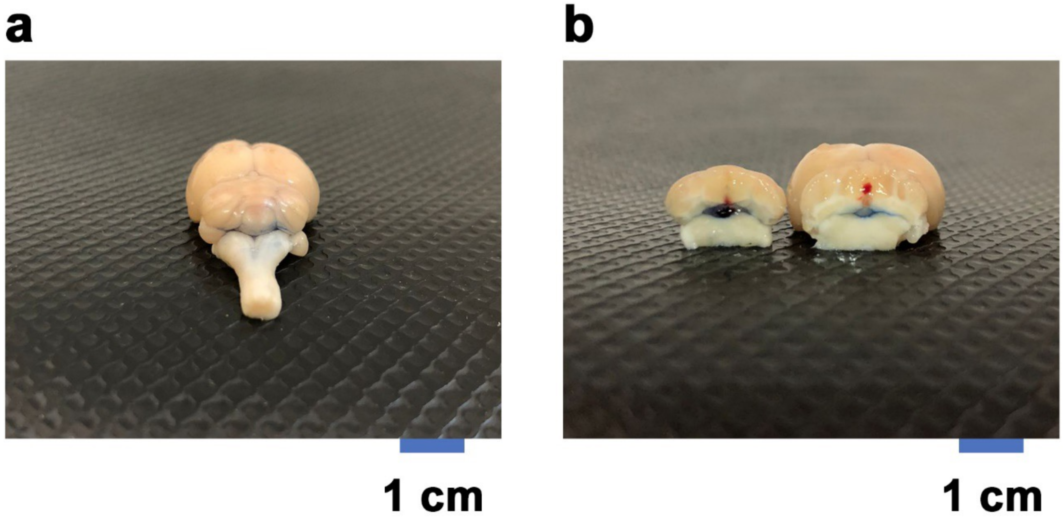

Figure S1. Representative brain for microinjection confirmation region in the 4th ventricle. (a) Brain with posterior view demonstrating the caudal portion with the presence of Evans blue. (b) Coronal section of the brain to verify the presence of dye in the 4th region.
